# Analysis of the expression levels of survivin and VEGF in patients with acute lymphoblastic leukemia

**DOI:** 10.3892/etm.2012.769

**Published:** 2012-10-26

**Authors:** MAN YANG, YONGQIANG LIU, SHUFANG LU, ZHIFEN WANG, RAN WANG, YOUMEI ZI, JINGDONG LI

**Affiliations:** 1 Departments of Hematology and; 2 Cardiovascular Surgery, The First Affiliated Hospital of Xinxiang Medical University, Weihui, Henan 453100, P.R. China

**Keywords:** survivin, vascular endothelial growth factor, expression, acute lymphoblastic leukemia

## Abstract

The purpose of this study was to investigate the association between an inhibitor of apoptosis (survivin) and vascular endothelial growth factor (VEGF) expression in patients with acute lymphoblastic leukemia (ALL) prior to and following chemotherapy. Reverse transcription (RT)-PCR and western blotting were employed to analyze survivin and VEGF mRNA and protein expression. Moreover, the concentrations of survivin and VEGF were determined by enzyme-linked immunosorbent assay (ELISA). Our data revealed that survivin and VEGF were overexpressed in patients with ALL prior to treatment, while survivin levels were significantly decreased following treatment. In addition, there was a positive correlation between survivin and VEGF concentrations in plasma. In conclusion, analysis of survivin and VEGF expression levels may improve the clinical diagnosis and treatment of ALL.

## Introduction

Survivin is a protein that inhibits apoptosis and regulates cell division (1). Vascular endothelial growth factor (VEGF) is a critical pro-angiogenic factor that induces proliferation and migration of endothelial cells within cancer vasculature (2). The expression levels of survivin and VEGF have been implicated to be associated with a wide range of clinical disorders, including cancer and cardiovascular diseases (3). For instance, survivin promotes DNA synthesis through the activation of CDK2/cyclin E and Rb gene phosphorylation and induces cancer cell proliferation and migration (4), while VEGF has been demonstrated to be a significant promoter of tumor neovascularity; positive associations between tumor VEGF expression and aggressiveness have been demonstrated in various types of cancer (5,6).

More recently, interactions of survivin with caspase and Fas were revealed to be associated with the development of leukemia (7). Based on these observations, we hypothesized that survivin and VEGF may be significant in acute lymphoblastic leukemia. In the current study, we carried out a systematic analysis of the expression of survivin and VEGF in patients with acute lymphoblastic leukemia (ALL) and performed a quantitative analysis of survivin and VEGF to discern whether they play a significant role in ALL.

## Materials and methods

### Study design and patients

Between 2011 and 2012, a total of 40 patients with ALL and 40 healthy controls, matched for age and body mass index (BMI), were recruited in this study. The selection for these patients was in accordance with the following criteria: i) a clinical and bone marrow diagnosis of ALL; ii) not receiving any chemotherapy, radiotherapy or anticancer drugs; iii) not previously treated for this disease. Patients who had a family history of autoimmune diseases or cardiovascular diseases were excluded.

The patients then underwent the following chemotherapy: idarubicin, 8–10 mg/(m^2^ per day) for three days; cytosine arabinoside, 100 mg/(m^2^ per day) for five days; and etoposide, 75–100 mg/(m^2^ per day) for five days. For comparison purposes, we considered that there were three groups: the 40 patients prior to treatment were defined as Group A; these patients following chemotherapy were defined as Group B; and the healthy controls were defined as Group C. The study was approved by the ethics committe of The First Affiliated Hospital of Xinxiang Medical University and informed consent was obtained from all patients.

### Reverse transcription (RT)-PCR

Total RNA was prepared using the Qiagen RNeasy Kit (Qiagen Inc., Valencia, CA, USA) and RT-PCR was performed using the SuperScript III One-Step RT-PCR kit (Invitrogen Life Technologies, Carlsbad, CA, USA) according to the manufacturer’s instructions. The specific primer pairs are as follows: for survivin, 5′-GGA CCA CCG CAT CTC TAC ATT-3′ (forward) and 5′-AGA AGA AAC ACT GGG CCA AGT C-3′ (reverse); for VEGF, 5′-TTG CTG CTC TAC CTC CAC-3′ (forward) and 5′-AAT GCT TTC TCC GCT CTG-3′ (reverse); and for β-actin, 5′-GCT CAC CAT GGA TGA TGA TAT C-3′ (forward) and 5′-GCC AGA TTT TCT CCA TGT CGT C-3′ (reverse).

### Western blot analysis

We further obtained 5-ml blood samples from the patients and healthy controls, mixed the blood (1 ml) with 5X loading buffer and then incubated the samples at 100°C for 80 min. All the samples were analyzed using the standard western blot method according to the manufacturer’s instructions (Biyuntian, Inc., Haimen, Jiangsu, China). For survivin, VEGF and β-actin measurement, we used the monoclonal antibody specific for the respective protein (BioVision, Inc., San Francisco, CA, USA), at a dilution ratio of 1:1,000.

### Determination of survivin and VEGF concentrations in plasma

After collecting the blood samples, we immediately placed them into sterile EDTA test tubes and centrifuged at 1,500 × g for 20 min at 4°C to collect plasma. The plasma was stored at −70°C until assayed. The concentrations of survivin and VEGF were analyzed by enzyme-linked immunosorbent assay (ELISA; Merck Millipore, Darmstadt, Germany) according to the manufacturer’s instructions.

### Statistical analysis

Statistical analysis was performed using SPSS 12.0 (SPSS Inc., Chicago, IL, USA). All values were expressed as the mean ± SD. Comparisons were made using the *χ*^2^ test or Fisher’s exact test. We also analyzed the correction between the concentrations of survivin and VEGF using Pearson’s two-tailed model. P<0.05 was considered to indicate a statistically significant result.

## Results

### Survivin and VEGF mRNA expression in patients with ALL

**Figure 1:**
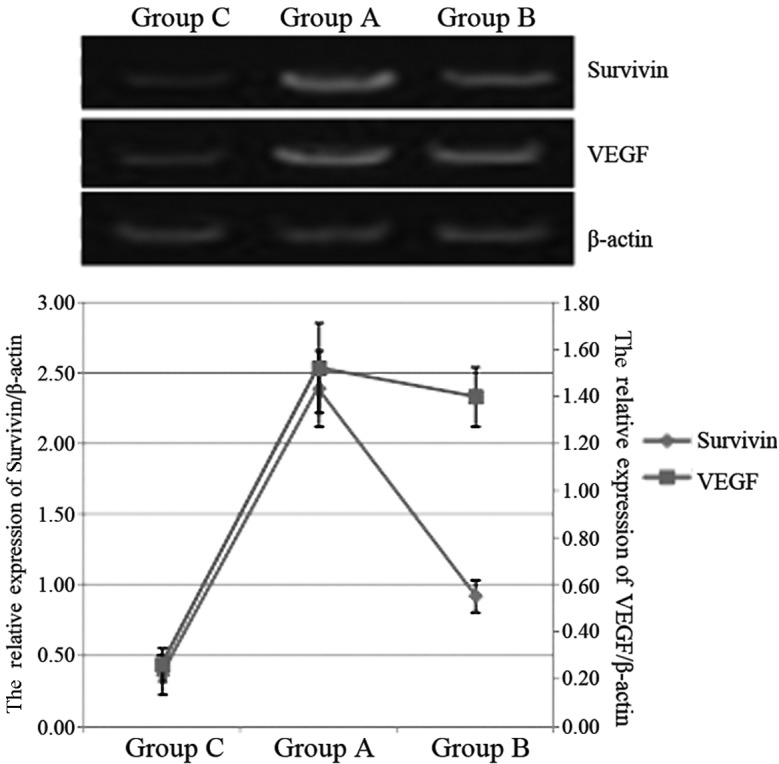
mRNA expression levels of survivin and vascular endothelial growth factor (VEGF) in patients with acute lymphoblastic leukemia (ALL).

Using an RT-PCR approach, we detected the mRNA levels of survivin and VEGF in patients with ALL. As shown in Fig. 1, in Group A, the survivin and VEGF mRNA levels were significantly higher than those in Group C (P<0.05), while the level of survivin mRNA in Group A was significantly lower than that in Group B (P<0.05). Compared with the VEGF mRNA levels of Groups B and C, that of Group A was not statistically significantly different (P>0.05).

### Expression of survivin and VEGF proteins in patients with ALL

**Figure 2:**
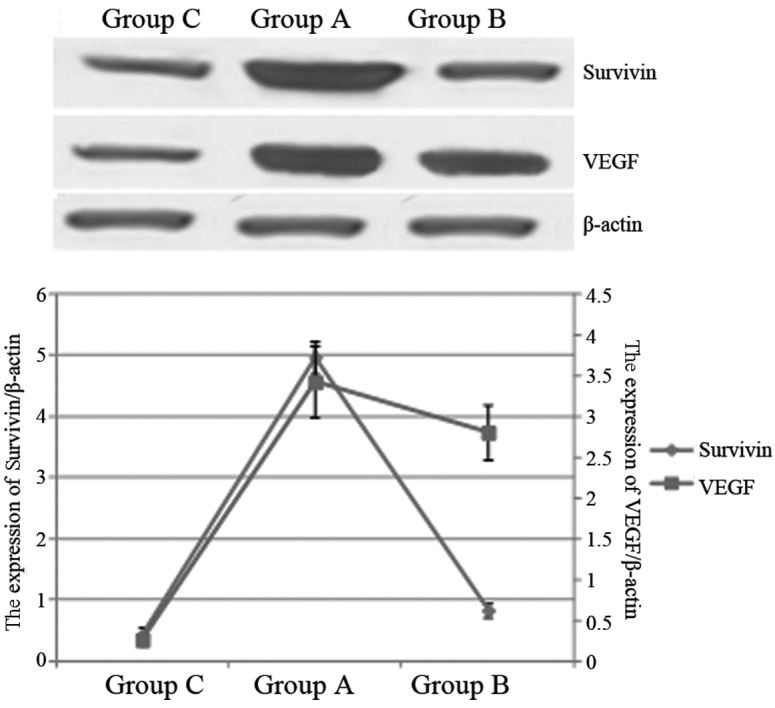
Survivin and vascular endothelial growth factor (VEGF) protein levels in patients with acute lymphoblastic leukemia (ALL).

We further used western blotting to analyze the survivin and VEGF protein expression in these groups. As shown in Fig. 2, the quantities of survivin and VEGF protein expressed in Group A were markedly higher than in Group C (P<0.05). Moreover, the survivin protein expression level in Group A was significantly lower than that in Group B (P<0.05), while there was no statistically significant difference between the VEGF protein expression levels in Groups A and B (P>0.05).

### Correlation between survivin and VEGF concentrations

**Figure 3:**
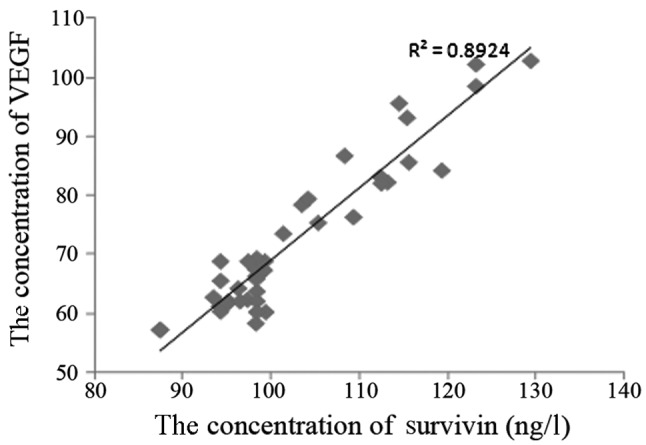
Correlation analysis of survivin and vascular endothelial growth factor (VEGF) concentrations in patients with acute lymphoblastic leukemia (ALL).

With the purpose of understanding the correlation between the concentrations of survivin and VEGF in plasma, we carried out an ELISA to analyze the concentrations. Fig. 3 shows the correlation between survivin and VEGF concentrations; it is clear that there is a significant correlation between survivin and VEGF (R^2^=0.8924).

## Discussion

ALL is the most common pediatric malignancy, affecting one in four children and adolescents younger than 20 years old. It accounts for 80% of all leukemia cases in children (8). Of the numerous variables that influence prognosis, genetic subsets, initial white blood cell count (WBC), age at diagnosis and early treatment response are the most significant (9). The clinical manifestations of ALL always include the symptoms of anemia, bleeding and organ infiltration. The standard treatment of ALL includes various phases (induction, consolidation/intensification and maintenance phases) in which combination chemotherapy, central nervous system (CNS) sanctuary therapy with intrathecal chemotherapy and high dose chemotherapy are administered. Consequently, multimodal therapy and enhanced supportive care have resulted in 5-year survival rates that approach 90% for those diagnosed at 14 years of age and younger (9)

Genetic studies have indicated that cytokines or intracellular transcription factors are involved in the the pathogenesis of ALL. Of these, survivin, a unique member of the inhibitor of apoptosis (IAP) protein family, is a cell-cycle regulator and its expression in cancer has been associated with cancer progression, drug resistance and shortened patient survival (10,11). Moreover, there are a growing number of studies concerning the association between survivin and ALL; Esh *et al*(12) reported that overexpression of survivin is a candidate parameter for determining poor prognosis in ALL patients, while knockdown of survivin improved the chemotherapeutic response in ALL models (13). A recent study suggested that survivin/NF-κB signal transduction pathways may influence leukemia treatment, while after treatment with drugs, survivin expression decreased significantly in the HL60/adr cell line (14). This is in accordance with the results revealed in the present paper. VEGF is a 45-kDa secretary glycoprotein responsible for endothelial cell differentiation, migration, proliferation, tubular formation and vessel assembly. Demacq *et al* indicated that polymorphisms in VEGF are associated with high relapse risk in ALL (15). In the present study, we demonstrated that the ALL patients’ VEGF mRNA expression levels were much higher than those of the control group. Moreover, we also identified that survivin and VEGF have a positive correlation by ELISA, but the molecular mechanism involving survivin and VEGF in the pathogenesis of ALL remains to be further experimentally investigated.
